# Mechanisms of Photoaging and Cutaneous Photocarcinogenesis, and Photoprotective Strategies with Phytochemicals

**DOI:** 10.3390/antiox4020248

**Published:** 2015-03-26

**Authors:** Ricardo Bosch, Neena Philips, Jorge A. Suárez-Pérez, Angeles Juarranz, Avani Devmurari, Jovinna Chalensouk-Khaosaat, Salvador González

**Affiliations:** 1 Department of Dermatology, Virgen de la Victoria University Hospital, Málaga 29010, Spain; E-Mails: ricardobosch@aedv.es (R.B.); jasuape@hotmail.com (J.A.S.-P.); 2 Dermatology and Medicine Department, University of Málaga, Málag 29071, Spain; 3 School of Natural Sciences, Fairleigh Dickinson University, 1000 River Road, Teaneck, NJ 07666, USA; E-Mails: avaniben@student.fdu.edu (A.D.); jovinna.ck323@yahoo.com (J.C.-K.); 4 Biology Department, Universidad Autónoma de Madrid, Madrid 28903, Spain; E-Mail: angeles.juarranz@uam.es; 5 Dermatology Service, Memorial Sloan-Kettering Cancer Center, New York, NY 10022, USA; E-Mail: gonzals6@mskcc.org; 6 Ramon y Cajal Hospital, Alcala University, Madrid 28034, Spain

**Keywords:** skin aging, skin cancer, ultraviolet radiation, signal transduction pathways, extracellular matrix, polyphenols

## Abstract

Photoaging and photocarcinogenesis are primarily due to solar ultraviolet (UV) radiation, which alters DNA, cellular antioxidant balance, signal transduction pathways, immunology, and the extracellular matrix (ECM). The DNA alterations include UV radiation induced thymine-thymine dimers and loss of tumor suppressor gene p53. UV radiation reduces cellular antioxidant status by generating reactive oxygen species (ROS), and the resultant oxidative stress alters signal transduction pathways such as the mitogen-activated protein kinase (MAPK), the nuclear factor-kappa beta (NF-κB)/p65, the janus kinase (JAK), signal transduction and activation of transcription (STAT) and the nuclear factor erythroid 2-related factor 2 (Nrf2). UV radiation induces pro-inflammatory genes and causes immunosuppression by depleting the number and activity of the epidermal Langerhans cells. Further, UV radiation remodels the ECM by increasing matrixmetalloproteinases (MMP) and reducing structural collagen and elastin. The photoprotective strategies to prevent/treat photoaging and photocarcinogenesis include oral or topical agents that act as sunscreens or counteract the effects of UV radiation on DNA, cellular antioxidant balance, signal transduction pathways, immunology and the ECM. Many of these agents are phytochemical derivatives and include polyphenols and non-polyphenols. The flavonoids are polyphenols and include catechins, isoflavones, proanthocyanidins, and anthocyanins, whereas the non-flavonoids comprise mono phenolic acids and stilbenes. The natural sources of polyphenols include tea, cocoa, grape/wine, soy, pomegranate, and *Polypodium leucotomos*. The non-phenolic phytochemicals include carotenoids, caffeine and sulphoraphance (SFN). In addition, there are other phytochemical derivatives or whole extracts such as baicalin, flavangenol, raspberry extract, and *Photomorphe umbellata* with photoprotective activity against UVB radiation, and thereby carcinogenesis.

## 1. Introduction

Premature skin aging and development of malignant cutaneous tumors, melanoma and non-melanoma, are interrelated issues that are increasingly important problems in the field of dermatology. Skin aging is important aesthetically, whereas skin cancer is a direct threat to the health of the patient. Hence, research aimed at providing knowledge in these areas has increased exponentially to develop preventive and therapeutic approaches.

We review the mechanisms of photoaging and photocarcinogenesis, the photoprotective strategies, and the phytochemicals that can provide photoprotection. The photoaging and photocarcinogenic mechanisms are predominantly the effect of solar ultraviolet (UV) radiation that induces reactive oxygen species (ROS) and alters DNA/cellular homeostasis, which together alter signal transduction pathways and inflammatory cascade and induce immunosuppression and extracellular matrix (ECM) remodeling. The photoprotective strategies include the blockade of UV photon incidence, DNA repair, removal of ROS (antioxidant), anti-inflammation, and immunomodulation. The photochemical derivatives that are effective for these photoprotective strategies are polyphenols, flavonoids and non-flavonoids, non-phenolic derivatives, and whole plant extracts.

## 2. Mechanisms of Photoaging and Photocarcinogenesis

Aging is a natural process leading to the progressive deterioration of the organs and its resultant clinical and histological changes. A primary cause is the imbalance between ROS production and their neutralization by natural antioxidant systems, which generates oxidative stress. ROS promote peroxidation of the lipid components of the cell membrane, alter the structure and function of several enzymatic systems, and promote carbohydrate oxidation. Incident UV radiation is the predominant cause of the oxidative stress in the skin and the histological differences between UV radiation exposed and non-exposed skin areas. The whole array of changes caused by UV radiation in exposed skin is termed photo aging, whereas changes from other factors that contribute to aging, such as metabolic or hormonal, are termed “chronologic” or “intrinsic” aging [1,2,3,4].

Cancer is a disease caused by the alteration of certain genes, resulting in uncontrolled cellular proliferation and loss of normal control mechanisms to inhibit such growth. Its development begins at the cross roads between genetics and environment, the latter being more important in certain types of cancer, e.g., skin tumors. The chief promoter of skin cancer, which underlies the use of the term photo carcinogenesis, is sun radiation; although other factors may contribute, such as viruses or chemicals.

**Figure 1:**
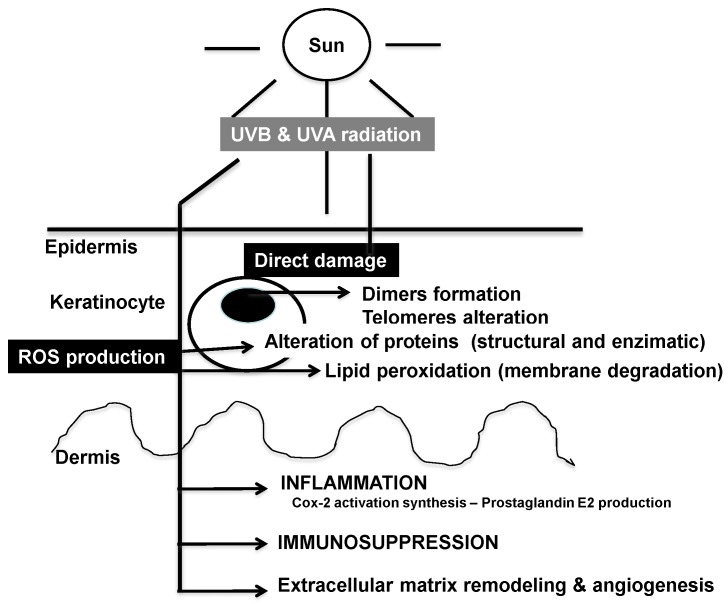
Summary of the major deleterious effects of sun-generated ultraviolet (UV) radiation in skin.

The photoaging and photocarcinogenic mechanisms through UV radiation induced ROS and DNA damage, and the resultant cellular damage, inflammation, immunosuppression and ECM remodeling/angiogenesis are illustrated in Figure 1.

### 2.1. Effects of Solar Ultraviolet Radiation

Photon energy carried in UV (particularly UVB at 280–315 nm, and UVA at 315–400 nm) induces alterations that accumulate and promote the majority of the typical manifestations of skin aging and cancer. UVB makes up only 5% of the UV radiation that reaches the surface of earth and has little penetrance, but it displays great biological activity. UVA makes up the remaining 95% of incident light and is more penetrating, promoting photo aging. However, UVA carries less energy and therefore promote carcinogenesis to a lower extent than UVB [5].

The main effects of acute and chronic exposure to UV radiation are DNA damage, inflammation and immunosuppression. These effects are direct as well as indirect due to ROS production. The ROS are particularly harmful in that they destabilize other molecules and promote chain reactions that damage biomolecules rapidly, such as telomere shortening and deterioration, mitochondrial damage, membrane degradation and oxidation of structural and enzymatic proteins [4].

UV radiation directly and through ROS participates in the three stages of the carcinogenic process [6,7,8]. During initiation, it produces genetic damage through direct effect on the DNA or by activating other factors [9,10]. In the promotion stage, it favors the proliferation of malignant cells by inhibiting the mechanisms of immune controls and promoting genomic instability [11]. Finally, it also boosts progression and dissemination of tumors by promoting protease release and angiogenesis [12,13].

### 2.2. DNA and Cellular Homeostasis

UV radiation, particularly UVB, alters DNA by promoting the formation of thymine-thymine dimers and pyrimidine-pyrimidone dimers, and generates ROS [14,15,16]. The thymine-thymine dimers are particularly crucial when they affect the tumor suppressor gene p53. Mutated p53 appears in skin displaying chronic sun damage, actinic keratosis and skin cancer [17]. The p53 mutation make cells resistant to apoptosis and the cells enter mitosis without having undergone DNA repair [18]. Further, the ROS participate in p53-independent apoptotic pathways [19].

### 2.3. Signal Transduction Pathways

The predominant pathways regulated by photooxidative stress include the mitogen-activated protein kinase (MAPK), the nuclear factor-kappa beta (NF-κB)/p65, the JAK/STAT (Signal Transduction and Activation of Transcription) and the nuclear factor erythroid 2-related factor 2 (Nrf2) [20,21]. The activation of MAP kinase pathway, through the receptor tyrosine kinase, results in the activation of transcription factor activator protein-1 (AP-1) that activates expression of MMPs and is comprised of the extracellular signal-regulated kinase 1/2 (ERK1/2), c-Jun-N-terminal- kinase (JNK) and p38 proteins [21]. The JNK and p38 pathways play a major role in the UV radiation mediated increase in AP-1 and cyclooxygenase-2 (COX-2) expression, and are targets for chemoprevention of skin cancer [5]. The transcription factor Nrf2 regulates the expression of phase II key protective enzymes through the antioxidant-response element. Nrf2 and several of its target genes are significantly down-regulated, causing oxidative stress in human prostate cancer [22].

The NF-κB pathway is also activated by oxidative stress through the activation of cytoplasmic I-κB kinase. Active I-κB kinase phosphorylates and degrades I-κB, the inhibitor of NF-κB transcription factor [20]. The NF-κB activation is associated with UVA and UVB radiation mediated oxidative modification of cellular membrane components [3]. The release of NF-κB from its inhibitor (I-κB), results in the translocation of active NF-κB to the nucleus to activate the inflammatory cytokines and prostaglandins [20]. In general, inhibition of NF-κB by use of antioxidants, proteasome inhibitors, prevention of Ikb phosphorylation or expression of overactivated, mutant (Ikb) mitigate UV-induced damage (Reviewed in [21]).

### 2.4. Role of Mitochondria and Cellular Bioenergetics

Mitochondria are important players in the oxidative response. Mitochondrial DNA accumulates UV-induced mutations that track exposure to UV light (reviewed in [22]). UV alters mitochondrial function, including decreased O2 consumption and ATP production, which effects cellular processes such as cell migration and division (Reviewed in [23]). The mechanism of UV-induced mitochondrial dysfunction and toxicity includes interrelated steps of caspase activation, membrane depolarization and cytochrome C release (Reviewed in [24]). Overall mitochondrial dysfunction, in addition to the depletion of cellular energy required activities such as motility and DNA repair, increases the levels of oxidative stress from increased ROS production at mitochondrial complexes (reviewed in [25]). UV radiation targets Nrf2. Nrf2 is a master regulator of the antioxidant response as it controls the expression of several endogenous antioxidant systems, e.g., the enzymes involved in NADPH synthesis such as glucose 6-phosphate dehydrogenase, synthesis of thioredoxin such as thioredoxin reductase, and GSH, such as glutathione S-transferases and peroxidase (reviewed in [26]). Nrf2 also controls the bioavailability of mitochondrial respiratory (reviewed in [27]), underlining the role of bioenergetics in Nrf2-mediated antioxidant protection.

### 2.5. Inflammation Cascade

UV radiation induces pro-inflammatory genes. Inflammation is an important mediator of photoaging and photocarcinogenesis [28,29,30,31]. The inflammatory mediators are released from keratinocytes, fibroblasts, tumor cells, leukocytes, and the endothelial lining of blood vessels. The mediators include the plasma mediators (bradykinin, plasmin, fibrin), lipid mediators (prostaglandins, leukotrienes, and platelet activating factor), and the inflammatory cytokines [interleukin-1 (IL-1), IL-6, and tumor necrosis factor (TNF)-α]. The lipid mediators, COX-2 (cyclooxigenase-2) and prostaglandin E2 (PGE2) are also activated by ROS [32,33,34]. UV radiation also participates in the activation of the enzyme ornithine decarboxylase, which decreases the activity of different polyamines that regulate cell proliferation [35]. The inflammatory process triggers ROS and RNS (reactive nitrogen species), which generates peroxynitrite that triggers DNA deletion and rearrangement [36,37]. The processes of DNA repair, cell cycle and apoptosis are altered to favor tumor progression. Further, UV radiation alters the expression of transforming growth factor-b (TGF-β), which is the predominant regulator of the matrix metalloproteinases that remodel the extracellular matrix for skin photoaging and tumor dissemination [38].

### 2.6. Immunosuppression

**Figure 2:**
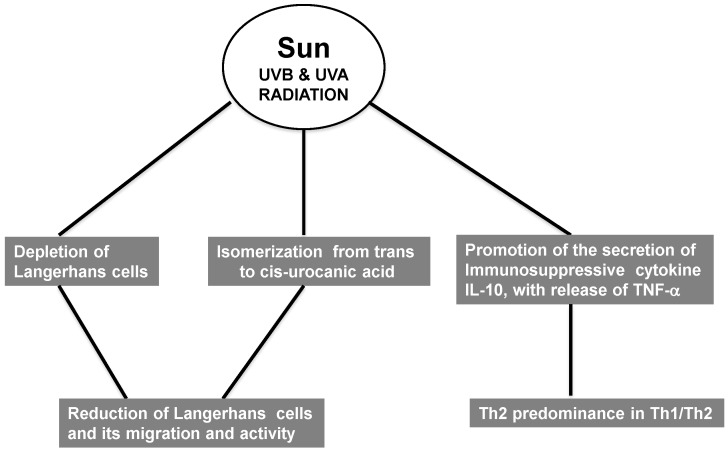
Immunosuppressive effects of UV radiation.

Immunosuppressed patients are more prone to develop tumors, likely due to decreased local immunosurveillance. UV radiation mainly decreases the cellular response, but the humoral response is also affected (reviewed in [39]). UV radiation depletes epidermal Langerhans cells (LC), which are crucial mediators of the cellular immune response due to their role in antigen presentation [40] (Figure 2). Importantly, UV radiation not only decreases the number of LC but also impairs their functions. e.g., migration and antigen presentation in lymph nodes. Whereas the former is not well understood mechanistically, impaired antigenic presentation is due to a loss of co-stimulatory molecules. e.g., B7. The main mechanism involved in this process is the isomerization of *trans*-urocanic acid to the *cis* form, which directly reduces LC migration and activity (reviewed in [41,42,43]). In addition, UV radiation promotes secretion of the immunosuppressive cytokines (IL-10). Il-10 is secreted by keratinocytes in response to *cis*-UCA or CPD. This cytokine is of particular interest because of its crucial role in immunosuppression in skin, not only induced by UV radiation but in other skin pathologies, e.g., melanoma, in which high IL-10 correlates with bad prognosis (reviewed in [44]). Also, depletion of LC and the proinflammatory microenvironment induced by UV causes influx of macrophages that activate regulatory T cells (TREG) and polarizes the Th1/Th2 response towards Th2 [37,38] (Figure 2). The role of the Th2 response in immunosuppression in response to UV light is likely related with the expression of IL12 by LC because IL-12 depletion skews T cell activation towards Th2 while promoting TREG activation (reviewed in [45,46,47]).

### 2.7. Extracellular Remodeling: Collagen, Elastin and Matrix Metalloproteinases Network

Collagen and elastin are the structural proteins of the ECM. The deterioration/remodeling of the collagen and elastin fibers facilitates angiogenesis and metastasis, and the damaged collagen and elastin proteins serve as additional sensitizers of photooxidative stress [48]. The specific properties of the different collagens are based on the lengths of the triple helical segments, interruptions to the triple helix, and amino acid modifications [28]. The elastin fibers that provide stretch-recoil properties to skin are composed predominantly of an elastin core (90%) surrounded by fibrillin microfibrils. The loss of proper elastin fibers occurs with the exposure of skin to UV radiation [4]. UV radiation also depletes the microfibrillar network in the epidermal-dermal layer and the dermis, which contributes to the aberrant elastic fibers [49].

The ECM proteolytic enzymes (MMPs/elastases) are produced by epidermal keratinocytes, fibroblasts, in the mediation of ECM remodeling and skin cancer [50,51,52]. Their basal levels increase with aging, and are further increased by environmental pollutants and UV radiation, resulting in the fragmentation of collagen and elastin fiber proteins for carcinogenesis. MMPs are categorized on the basis of the presence of AP-1 or TATA nucleotide sequences in the promoters into group I MMPs (MMP-1, 3, 7, 9, 10, 12, 13, 19, and 26) that contain TATA box and activator protein-1 (AP-1 site), group II MMPs (MMP-8, 11, 21) without the AP-1 site, and group III (MMP-2, 14, 28) without the TATA box and AP-1 site [53,54].

The transcription factor AP-1, stimulated largely by the MAPK pathway, stimulates the transcription of several MMPs that collectively degrade the ECM, such as MMP-1, MMP-2/9, and MMP-3 [3]. Further, AP-1 inhibits the transcription of type I collagen gene [28]. Hence, the damage to the ECM and tissue integrity is from the enhanced degradation of ECM by MMPs as well as the reduced expression of the structural ECM proteins. The pro- and active forms of MMPs are inhibited by the tissue inhibitors of MMPs or tissue inhibitor of matrixmetalloproteinases (TIMPs) [53,54]. The remodeling of collagen and elastin, for angiogenesis, metastasis, and tissue destruction, is largely from the increased expression or activation of MMPs and reduced expression of TIMPs [53,54].

## 3. Photoprotective Strategies

**Figure 3:**
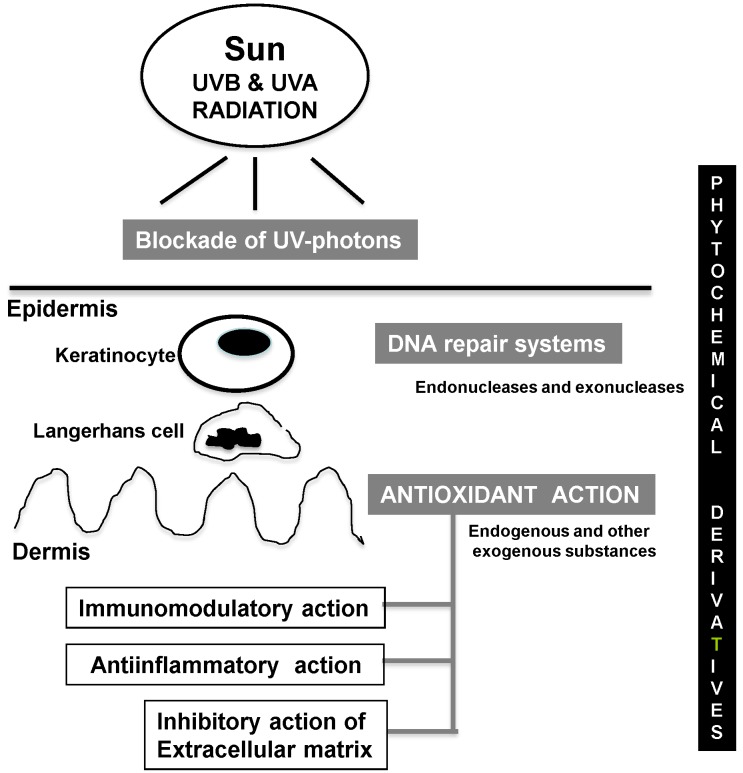
Major beneficial effects of phytochemical derivatives against UV-induced photodamage.

The photoprotective strategies to prevent and/or repair the deleterious effect of UV radiation leading to photoaging and photocarcinogenesis from direct blockade of UV photons, to counteracting the direct or indirect effects of UV radiation through DNA repair systems and antioxidants, are illustrated in Figure 3.

### 3.1. Blockade of UV Photon Incidence

The most obvious strategy to prevent the deleterious effects of UV radiation is to prevent its incidence on the skin; thus, physical blockers and screens are the most widely accepted and used countermeasures. Topical sunscreens can be divided into reflective and absorbing substances. Their use requires application of the correct amount, and frequent replenishment upon changing environment conditions, e.g., increased perspiration, water immersion, *etc.* Additional difficulties to their use include displeasing sensitivity, e.g., stickiness, aesthetic issues due to whitening, *etc.* Finally, complete blockade in cases of extreme photoprotection may lead to Vitamin D deficiency, which can promote carcinogenesis [55]. 

Sunscreens are the most important preventive measures against photoaging and photocarcinogenesis. In fact, proper use of sunscreens mitigates the chances of developing UV-induced skin cancer (reviewed in [56,57,58]). Some phytochemical derivatives fulfill this function, administered either topically or systemically, which increases adherence to the application regime as well as its uniformity.

### 3.2. DNA Repair Systems

Oxidative DNA damage is repaired by multiple, overlapping DNA repair pathways. Two major mechanisms exist to repair oxidatively induced DNA lesions: base-excision repair (BER) and nucleotide-excision repair (NER). In BER-mediated repair, DNA glycosylase usually detects the damaged base and mediates base removal prior to nuclease, polymerase, and ligase proteins bridging the gap and completing the repair process. On the other hand, NER-mediated repair recognizes base lesions that distort the helical structure. The damaged base is excised as a 22–30 base oligonucleotide resulting in single-stranded DNA that is repaired by proteins such as DNA polymerase before proceeding to ligation. An endogenous enzymatic system corrects and repairs the formation of cyclobutane pyrimidine dimers. The absence of the DNA repair systems produces diseases such as xeroderma pigmentosum, and underlies a large percentage of skin cancers in the general population.

Different preparations containing DNA repair enzymes have been assayed and shown to decrease DNA damage caused by UV radiation [59,60]. Among these, photoliase promotes a process termed DNA photo-reactivation that repairs T-T, cyclobutane pyrimidine dimers and 6-4 photoproducts [61]. Another preparation containing T4 endonuclease N5 (Dimericine) promotes DNA repair in xeroderma pigmentosum patients [62].

### 3.3. Antioxidant Activity

Oxidative stress is a key process underlying photo aging and photo carcinogenesis [63]. Endogenous systems to prevent its deleterious effects include enzymes, e.g., superoxide dismutase, catalase, ceruloplasmin, transferrin, *etc.*, and other substances obtained from the diet, e.g., Vitamin E (α-tocopherol), Vitamin C (ascorbic acid) and carotenoids (β-carotene). Non-melanoma skin cancers display a significant decrease of the enzymatic antioxidant systems [64]. However, whether exogenous supplementation of non-enzymatic antioxidants, e.g., Vitamin E, C or carotenes, is useful to prevent photocarcinogenesis is currently controversial [65,66,67,68]. There is disparity in the parameters evaluated, including erythema, immunosuppression, photo aging or tumor formation, and the lack of homogeneity may account for the controversy [69,70]. A largely beneficial substance to prevent skin cancer is Vitamin A (retinol, derived from β-carotene) and its derivatives (isotretinoin and acitretin) [71,72,73].

### 3.4. Anti-Inflammatory Action

Aspirin (acetyl salicylic acid) and other NSAIDs (non-steroidal anti-inflammatory drugs), e.g., indomethacin, piroxicam, sulindac, diclofenac, are useful to decrease the incidence of skin tumors and to treat actinic keratosis [74,75]. These molecules decrease prostaglandin production by inhibiting COX-1 and COX-2 [76]. Colecoxib is another inhibitor specific of COX-2 [77]. Several studies support the anti-inflammatory effect of different phytochemicals [78,79].

### 3.5. Immunomodulatory Action

Another strategy against photo carcinogenesis is to revert immunosuppression induced by UVB. For example, supplementation with Vitamin B3 (niacin) reduced the immunosuppressive effect of UVB [80]. Other phytochemicals decrease immunosuppression, e.g., by preventing Langerhans cell depletion [81].

### 3.6. Inhibitory Activity of ECM Remodeling

An emerging strategy against photo aging is to prevent the changes induced by UV, particularly UVA, in the dermis. These changes include alterations of the extracellular matrix proteins that form fibrillar structures, e.g., collagen and elastin. Metalloproteinase and elastase expression and/or activation underlie these alterations, which also promote angiogenesis and tumor progression. Several phytochemicals orally and/or topically administered provide protection against these changes [82].

## 4. Photoprotective Activity of Phytochemical Derivatives

A myriad of extracts or isolated/purified substances from different parts of plants, including roots, leaves, flowers, seeds, *etc.*, have been traditionally used to prevent and treat skin cancer [2]. Although some of these substances have been used topically, its route of administration is mainly oral, as food supplements, concentrates and purified extracts. Many of these substances contain active principles of the polyphenol group (antioxidants), or other antioxidants with diverse chemical structures [83]. The active phytochemicals or the extracts of their sources have become major photoprotective strategies. Although they mainly function as antioxidants, they also display anti-inflammatory and immunomodulatory activity and also control dermal extracellular matrix remodeling.

### 4.1. Polyphenols

Polyphenols are chemicals characterized by the presence of more than one phenolic group (a hydroxyl group bound to an aromatic ring) per molecule. Their intrinsic antioxidant function resides in the hydroxyl (−OH) group that, bound to the aromatic ring, act as a hydrogen or an electron donor, giving it to a free radical or other reactive species. This underlies the inhibition of ROS and ROS mediated damage on DNA, proteins and lipids; production of inflammatory cytokines; and the activation of the signal transduction pathways such as mitogen activated protein kinase and nuclear factor kappa-B (NF-κB)/p65 that regulate transcriptional activity [84].

The typical classification of these molecules takes into account the number and type of phenolics, which determine their biological properties. According to this, polyphenols are either flavonoids (the most numerous) or non-flavonoids, appearing in numerous plants (Table 1). In addition to their antioxidant capability, some of them display metal (Cu and Fe) chelating properties, thereby preventing the Fenton reaction, which involves formation of free radicals from hydrogen peroxide (H_2_O_2_). Non-flavonoids comprise mono phenolic acids and alcohols, benzoic and cinnamic acid and stilbenes.

#### 4.1.1. Flavonoids

The flavonoids include catechins, isoflavones, proanthocyanidins, and anthocyanins (Table 1).

The catechins are mainly present in tea leaves; they contain a pyrocatechol group and comprise of the following: catechin, epicatechin, galactocatechin, epicatechingallate and epigallocatechin-3-gallate. In humans, their topical administration decreases UV-induced changes in epidermis, specifically increased p53 expression and apoptosis [85].

**Table 1:** Main sources of polyphenols with antioxidant activity.

Polyphenol	Major Sources
**Flavonoids**	
Catechins: catechin, epicatechin, galactocatechin, epicatechingallate, epigallocatechin-3-gallate	Tea
Isoflavones: Genistein	Soy
Sylimarin	Thistle
Proanthocyanidins (tannins)	Grapeseed
Anthocyanins	Pomegranate
**Non-flavonoids**	
Phenolic acids	Grape & derivatives
Benzoic acids: Galic acid	Tea
Cinnamic acids	*Polypodium leucotomos*
Stilbene	Grape & derivatives
Resveratrol	Nuts, peanuts

The isoflavones contain a phloroglucinol group. The most well-known isoflavones are genistein, derived from soybean, and silymarin, derived from the milk thistle (*Silybum marianum*). Genistein exerts a photoprotective effect and halts skin photocarcinogenesis in animal models [86]. The major active principle of silymarin is silibinin. It is widely used as a liver protector, but it has been shown to be also photoprotective in animals due to ROS reduction as it decreases infiltration of CD11b+ lymphocytes in UV-irradiated areas [87].

The proanthocyanidins are also known as condensed tannins; this is a group of substances widely represented in grape seeds. An extract of grape seeds prevents tumor induction in response to UV radiation in mice. These effects are likely due to their antioxidant and anti-inflammatory properties [88,89].

The anthocyanins are water-soluble blue dyes and confer this color to leaves and fruits and seeds, e.g., grapes, to protect them against solar radiation. It has an important role for the color/appearance of wine.

#### 4.1.2. Non-Flavonoids

The non-flavonoids include phenolic acids and stilbene (Table 1). The phenolic acids include benzoic, galic, and cinnamic (caffeic, ferulic and p-cumaric) acids. They appear in wine (more in red wine) and in tea. They exhibit antioxidant and anti-neoplastic properties. Caffeic acid, not related to caffeine, belongs to the hidroxycinnamic group and is widely present in all plants. It protects against UVA-induced photo damage [90].

Stilbene represents anti-microbial substances secreted by plants. The most important is glycosylated *trans*-resveratrol (in grapes, grape juice and wine, peanuts and others). It is a good antioxidant with anti-aging and anti-photocarcinogenic properties in animals [91,92].

#### 4.1.3. Natural Sources of Polyphenols

The polyphenols are part of a normal diet, occurring in vegetables, fruits, beans and cereals. They are often used as concentrated dietary supplements obtained from common or uncommon vegetables. The topical use of polyphenols depends on their lipophilic or hydrophilic nature. In general, polyphenols are hydrophilic and hardly penetrate the epidermal barrier (an exception is silymarin). The steps to address this have mainly been the improvement of penetrance by iontophoresis or use of liposomes. The natural sources of polyphenols include tea, cocoa, grape/wine, soy, pomegranate, and *Polypodium leucotomos* (Table 1).

Tea is the second most consumed liquid in the world, after water. It is an infusion of the leave of *Camellia sinensis*. Its name depends on the main features of the plant leaves used to brew the beverage, white (young leaves), green (non-oxidated), yellow or blue (medium oxidation) or black (high degree of oxidation, which increases its theaflavin content). Other denominations are geographical. Green tea is the most consumed tea around the world. It mainly contains catechins, particularly epigallocatechin-3-gallate and simple phenolic acids. It protects against sun damage, and anecdotal epidemiological evidence suggests that it reduces the development of certain tumors [93,94].

Cocoa extracts contain polyphenols, particularly catechins and proanthocyanidin flavanols, as well as smaller amounts of gallocatechin and epicagallo-catechin. In addition, cocoa contains large amounts of theobromine, a methylxanthine with ROS scavenging properties in skin upon UV treatment [95]. 

Grape, *Vitis vinífera*, contains numerous polyphenols in its seeds and grape peels. These pass to the fermented form, wine. Specifically, they contain antocyanins (which provide color) and other flavonoids, e.g., proanthocyanidins. Some of these include tannins that cause constipation. Grape/wine contains several non-flavonoid polyphenols, e.g., cinnamic acid and resveratrol, which are well-known photoprotectors. 

Soy, *Glycine maxi* (soybean plant), is enriched in proteins that are consumed in several forms, e.g., boiled bean pods, soybean cake, milk and sauce. It is high in protein content, which enables its use as meat or fish substitute. It also contains large amounts of genistein, which has photoprotective activity for the prevention/treatment of photoaging, and photocarcinogenesis [96,97].

Pomegranate extract contains anthocyanins, ellagitannins and hydrolyzable tannins. Its oral administration reduces UVB-induced carcinogenesis in mice [98]. 

*Polypodium leucotomos* extracts, from the tropical fern, contain a high concentration of antioxidant phenolic acids, e.g., caffeic and ferulic acids [99]. In addition, it contains monosaccharides, e.g., fructose and glucose, and many other components [88]. It is safe when administered orally and can undergo topical absorption. It displays great efficacy against photo aging and photo carcinogenesis [89]. It prevents lipid peroxidation, UV-induced membrane damage, transcriptional activation of proinflammatory AP1 and NF-κB factors, and induction of enzymes that generate nitric oxide [100]. It inhibits UV-mediated actin disarray and loss of cell-extracellular matrix focal adhesion and also prevents keratinocyte apoptosis [101,102,103,104]. It is also used as an adjuvant in PUVA (Psoralens + UVA) therapy to prevent the deleterious side effects of irradiation [105]. Its mechanism of action involves antioxidant, anti-inflammatory and immunomodulating activities [81,106,107,108]. In addition, it modulates metalloproteinase activity by inducing TIMP (tissue inhibitor of metalloproteinase) and induces elastin and collagen to counteract skin aging and photocarcinogenesis [109].

### 4.2. Non-Polyphenols with Photoprotective Sctivity

The non-phenolic phytochemicals include carotenoids, caffeine, and sulphoraphance (SFN) as well as several other whole extracts or components.

Carotenoids are pigments synthesized by plants and exhibit clear antioxidant action. They are mainly present in yellow or orange vegetables and fruits. They require additional fat for their absorption. B-carotene has been used as a photo protector in patients with pathological photosensitivity (porphyria and some others), but their sustained administration does not display any beneficial effect against non-melanoma cancer incidence compared to a control group [110]. Lycopene is a carotenoid most abundant in tomatoes. It is not a Vitamin A precursor, yet it protects against various skin alterations induced by UV radiation [111].

Caffeine is present in large amounts in coffee. Some epidemiological studies indicate that drinking large amounts of coffee decreases the incidence of skin cancer, particularly BCC (basal cell carcinoma) [112,113]. Caffeine underlies this effect since decaffeinated coffee does not replicate this finding [114]. The mechanism of this effect involves increased apoptosis of cells with defective DNA repair [115].

Sulphoraphane (SFN) is present largely in broccoli, extracts of which are metabolised into isothiocyanates. They main isothiocyanate from broccoli is SFN that has been shown to decrease UV-induced skin erythema in humans [116] and reduce the risk of skin cancer in mice [117]. Glucoraphanin, also known as SFN glucosinolate (SGS), is the precursor of SFN. It seems that SFN induces transcriptional activation of Nrf2. Other protective mechanisms in cells include inhibition of the activation of procarcinogens, disposal of damaged and potentially neoplastic cells by cell cycle arrest and apoptosis, and the suppression of inflammatory responses. 

Other phytochemical derivatives or whole extracts with photoprotective activity against UVB radiation are represented in Table 2 [118,119,120,121,122]. In addition, hypericin (Saint John Wort) can lower erythema in photodyanamic therapy, without being phototoxic [123].

Most of these studies have been carried out in animal models using topical or systemic administration.

**Table 2:** Other phytochemical derivatives with photoprotective activity against UVB radiation in mice (Murine model).

Substance and Origin	Activity	Reference
Topical “Baicalin”	Inhibition of Ki67, PCNA and COX-2 expression	[118]
Genus *Scutellaria*		
Oral “Flavangenol”	Reduction of Ki-67, and (8-OHdG)-positive cells and VEGF expression	[119]
French maritime pine bark extract		
Topical black raspberry extract	Reduction of edema, p53 levels and neutrophil activation	[120]
Topical *Photomorphe umbellata* extract	Inhibition of the hyperplastic reaction and p53-positive cells	[121]
Oral and topical Brown algae polyphenols	Inhibition of ciclooxygenase-2 activity and cell proliferation	[122]

## 5. Conclusions

We have reviewed the mechanisms of photoaging and photocarcinogenesis, the photoprotective strategies, and the phytochemicals that can provide photoprotection. The photoaging and photocarcinogenic mechanisms are primarily through UV radiation induced ROS and DNA damage and the resultant cellular damage, alterations in inflammatory/oxidative stress mediating signal transduction pathways, inflammation, immunosuppression and ECM remodeling/angiogenesis. The photoprotective strategies include the blockade of UV photon incidence, DNA repair through DNA repair enzymes, removal of ROS with antioxidant agents, and anti-inflammation/immunomodulation with anti-inflammatory agents. Many of these photoprotective strategies involve photochemical derivatives, including polyphenols (flavonoids and non-flavonoids), non-phenolic derivatives and whole plant extracts. An increase in the blockage of UV radiation as well as the strengthening of cellular antioxidant balance will reduce the incidence of photoaging and photocarcinogenesis.
